# Circular RNAs signature predicts the early recurrence of stage III gastric cancer after radical surgery

**DOI:** 10.18632/oncotarget.15288

**Published:** 2017-02-11

**Authors:** Yan Zhang, Jin Li, Jiang Yu, Hao Liu, Zhiyong Shen, Gentai Ye, Tingyu Mou, Xiaolong Qi, Guoxin Li

**Affiliations:** ^1^ Department of General Surgery, Nanfang Hospital, Southern Medical University, Guangzhou, China

**Keywords:** circular RNA, gastric cancer, prediction, recurrence

## Abstract

Tumor recurrence is usually detected within one year after radical resection of stage III gastric cancer. This study aimed to establish the expression profile and determine potential circular RNAs (circRNAs) and predict the early recurrence of stage III gastric cancer. We identified 46 differently expressed circRNAs between cancer and adjacent normal tissues through circRNA microarray. We further screened eight indicators related to early recurrence. We subsequently divided the remaining cases into two cohorts. qRT-PCR results demonstrated a significantly different outcome between low and high expressed groups of four circRNAs in the training cohort. We then constructed a four-circRNA-based classifier to evaluate the risk of early recurrence and distinguished patients with a high risk from those with a low risk. The areas under the receiver operator characteristic curve (ROC) of this classifier were 0.763 and 0.711 in the two cohorts, respectively. A new formula could be established by combined the circRNA classifier with TNM stages. The areas under the ROC curve were 0.866 and 0.818 of the two cohorts, respectively. Our study suggested that this four-circRNA-based classifier yielded a predictive ability to the early recurrence of stage III gastric cancer after radical surgery.

## INTRODUCTION

Gastric cancer is a common malignant tumor with the fourth highest occurrence and the third leading cause of death worldwide [1]. Although the treatment of gastric cancer has gradually improved, the mortality rate remains high [2]. Among patients with surgically resectable gastric cancer, approximately 30%–50% suffer from stage III cancer [3, 4]. Majority of these patients receive adjuvant radiotherapy and chemotherapy after they undergo surgical resection; however, more than 30% of these patients develop recurrence or distant metastasis with a 14-month median recurrence-free survival time [5, 6]. In general, T stage, N stage, differentiation type, and Lauren's classification are used to determine the possibility of postoperative recurrence for stage III gastric cancer. However, these clinical predictors cannot achieve a good sensitivity and specificity.

Advancements in molecular medicine help predict the possibility of the recurrence of stage III gastric cancer with molecular markers effectively. In a series of molecular markers, circular RNA (circRNA) provides several advantages. CircRNAs are closed-loop RNAs produced through an end-to-end formation of RNA transcription fragment during transcription. These RNAs have been investigated for more than 40 years [7], but they didn't attract enough attention until they have been extensively explored in the recent years. circRNAs are highly expressed RNAs in many tissues [8–10]. circRNAs often share the same transcript with the corresponding linear isomers, but they are formed through different splicing mechanisms [11, 12]. Previous studies demonstrated that circRNAs existed widely in all kinds of organizations [11, 13, 14]. In certain cancers, circRNA exhibits abnormal expression levels in epithelial tumors, such as laryngeal cancer and digestive system cancers [15–17], and in stromal tumors, such as gliomas [18]. Additionally, circRNA plays a strong regulatory function in carcinoma [19, 20]. Compared with mRNA, circRNA is composed of a ring structure that is hard to break down. Thus, circRNA remains stable and serves as a convenient tool for qRT-PCR measurements.

Our study aimed to establish the expression profile of stage III gastric cancer through circRNA microarray chip detection. Our results revealed the potential role of circRNAs in early gastric cancer recurrence. We also aimed to develop a multi-circRNA-based classifier for the prediction of the recurrence probability of stage III gastric cancer in a training cohort. We then validated the sensitivity and specificity of this classifier in a testing cohort.

## RESULTS

### Circular RNA microarray analysis

**Figure 1:**
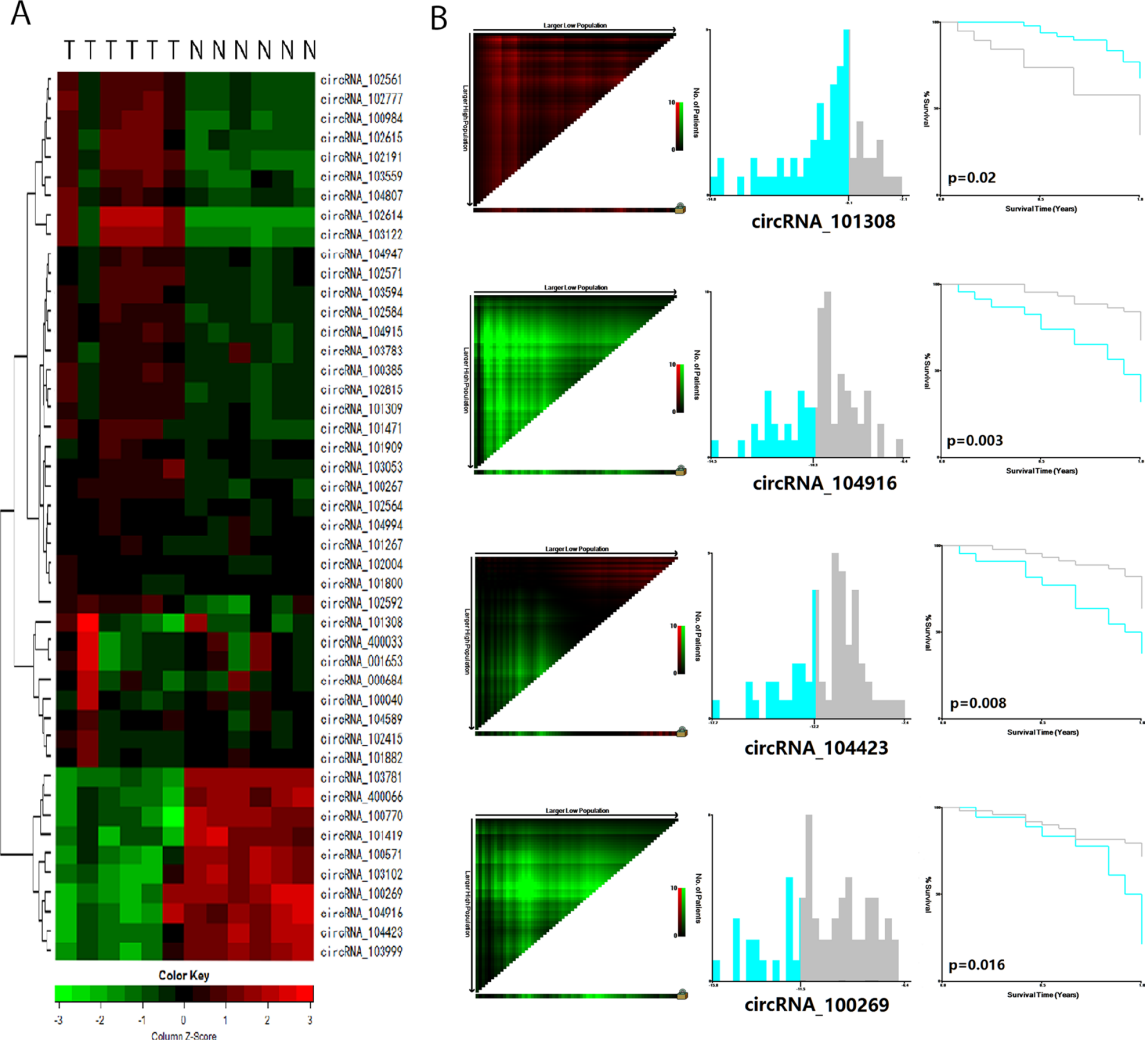
CircRNAs predict the early recurrence of stage III gastric cancer after radical surgery (**A**) Cluster analysis of 46 differentially expressed circRNAs in 6 paired tumor tissue and adjacent normal mucosa tissue. Every row represents an individual gene, and each column represents an individual sample. Pseudocolours indicate transcript levels from low to high on a log 2 scale from -3 to 3, ranging from a low relative expression (green) to a high relative expression (red); (**B**) The X-tile plots of 4 statistically significant different expressed circRNAs in the training cohort. Coloration of the plot represents the strength of the association at each division, ranging from high (bright, red or green) to low (dark, black). Green represents direct association between marker expression and survival, whereas red represents inverse association.

Of the 3,071 expressed circRNA indicators, 46 indicators revealed different expression levels in the six pair of tumors and adjacent normal mucosal specimens (Supplementary Figure 1). The result of cluster analysis was shown in Figure 1A. Among these circRNAs, eight indicators yielded different expression levels between the two tumor tissues from patients who developed recurrence within 1 year and the four other tumor tissues (Table 1). The levels of hsa_circRNA_101308, hsa_circRNA_400033, and hsa_circRNA_001653 in the recurrent cancer tissues were significantly higher than those in the non-recurrent cancer tissues. By contrast, the levels of hsa_circRNA_103781, hsa_circRNA_104423, hsa_circRNA_103999, hsa_circRNA_104916, and hsa_circRNA_100269 in the recurrent cancer tissues were significantly lower than those in the non-recurrent cancer tissues.

**Table 1:** Dysregulated circRNAs selected by microarray

CircRNAs	FC between tumor and adjacent mucosa	*P*-value	Regulation	FC between recurrence cases and non-recurrence cases	Regulation
**circRNA_101308**	5.1072551	0.019628228	up	11.34952005	up
**circRNA_400033**	3.0423671	0.030028469	up	5.896942224	up
**circRNA_001653**	3.2103063	0.017975042	up	5.291329627	up
**circRNA_103781**	3.7102396	0.012408294	down	4.044226970	down
**circRNA_104423**	3.5205997	0.003324673	down	4.454156227	down
**circRNA_103999**	3.0443244	0.010797271	down	4.638867676	down
**circRNA_104916**	3.4514459	0.025033225	down	9.911525363	down
**circRNA_100269**	4.7765489	0.011625393	down	8.815309110	down

### Circular RNA signature classifier selected by qRT-PCR

We first detected the level of the 8-chip-selected circRNAs in the training cohort. Among the 8 circRNAs, 7 were expressed, whereas hsa_circRNA_400033 was not expressed. In the training set, the survival curve were significantly different between the high-expressed and low-expressed groups of 4 circRNAs (*p* ≤ 0.05), and the cutoff line between high-expressed and low-expressed groups was calculated by X-tile software (Figure 1B). Univariate and multivariate cox regression analyses showed that 4 circRNAs, namely, hsa_circRNA_101308, hsacircRNA_104423, hsa_circRNA_104916, and hsa_circRNA_100269, were significantly different between the high and low expression groups in the training cohort (Supplementary Table 1).

### Construction of four-circRNA-based classifier

**Figure 2:**
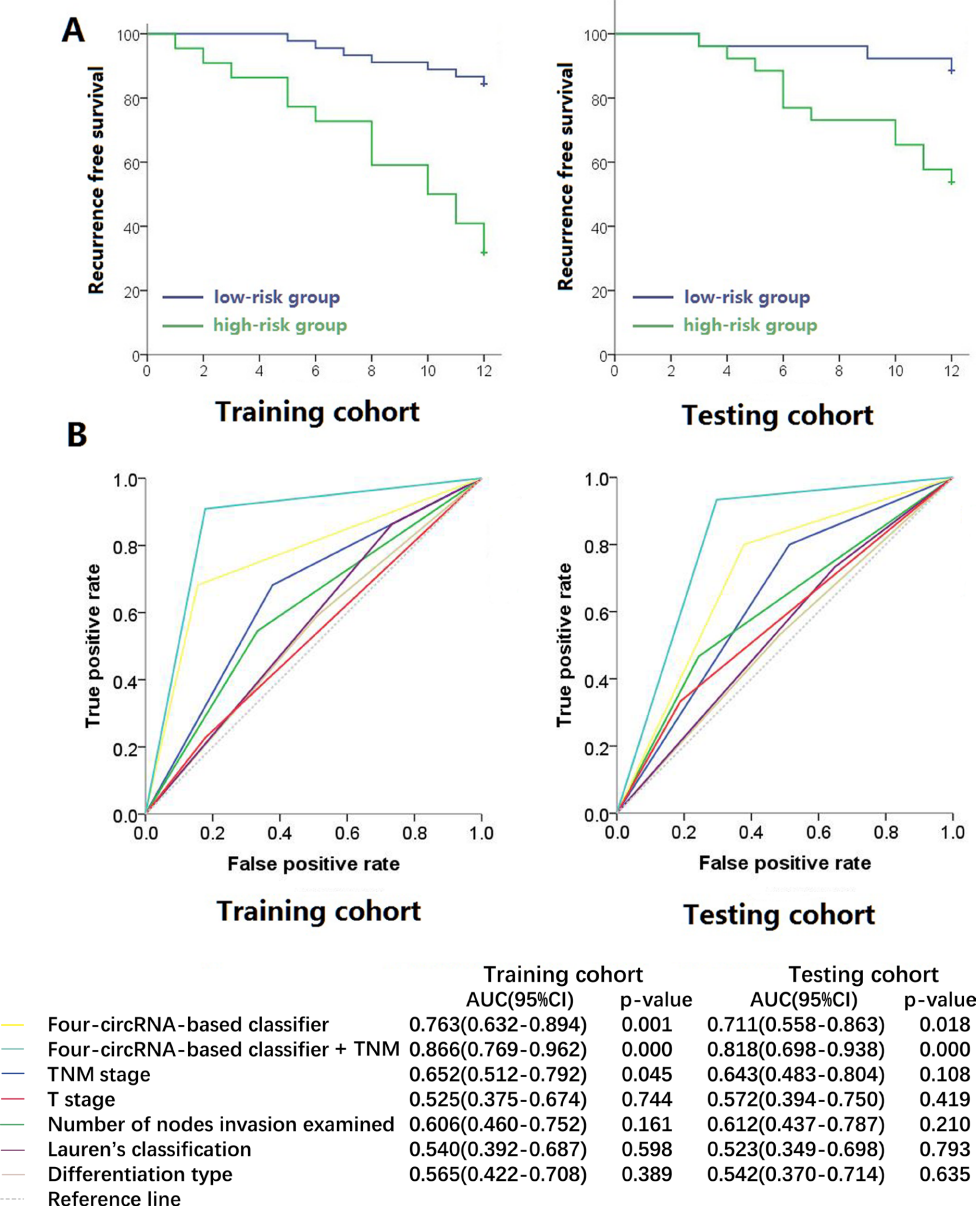
Kaplan-Meier survival (**A**) and Receiver operator characteristic curves (**B**) of training cohort and testing cohort. Data (95% CI) are area under curve at 12 months to assess predictive accuracy, and *p* values were calculated using the log-rank test.

We then constructed a four-circRNA-based classifier. The risk score formula of recurrence probability was established on the basis of the individual expression level of the four circRNAs: risk score = (1.434 × status of hsa_circRNA_101308) − (0.917 × status of hsa_circRNA_104423) − (1.042 × status of hsa_circRNA_104916) − (1.201 × status of hsa_circRNA_100269), where a high expression status is equal to 1 and a low expression status is equal to 0 in this formula. The cutoff score was “−1” calculated by X-tile software. Patients were included in the high-risk group when their risk scores were higher than the cutoff score. Otherwise, patients were included in the low-risk group. Using this classifier, we could distinguish gastric cancer patients with a low risk of early recurrence from those with a high risk. Recurrence probability was also significantly different between the groups in each cohort (Figure 2A). In the training cohort, the recurrence probabilities of low- and high-risk groups were 15.6% and 68.2%, respectively (hazard ratio [HR] = 6.248, 95% CI = 2.534–15.403, *p* = 0.000069). Similar results were observed in the testing cohorts (HR = 4.886, 95% CI = 1.375–17.359, *p* = 0.014). The HR of the clinical characteristics and circRNAs with recurrence are shown in Table 2. The areas under the receiver operator characteristic (ROC) curve were 0.763 (95% CI = 0.632–0.894) and 0.711 (95% CI = 0.558–0.863) in the two cohorts, respectively (Figure 2B). The Harrell C-indices of the two cohorts were 0.722 (95% CI = 0.597–0.847) and 0.681 (95% CI = 0.538–0.823), respectively.

**Table 2:** Hazard ratio of clinical characteristics and circRNAs with recurrence free survival

	Training set		Testing set	
	HR(95%CI)	*P* value	HR(95%CI)	*P* value
**Sex (male vs female)**	0.669 (0.247–1.815)	0.430	0.985 (0.314–3.095)	0.980
**Age (older than 65 years vs 65 years or younger)**	1.510 (0.511–4.461)	0.456	0.221 (0.029–1.679)	0.144
**TNM stages (IIIA and IIIB vsIIIC)**	2.850 (1.160–7.000)	0.022	3.045 (0.859–10.800)	0.085
**T stage (T2-T4a vs T4b)**	1.288 (0.475–3.492)	0.619	1.956 (0.668–5.730)	0.221
**Number of nodes invasion examined (less than 16 vs 16 or more)**	2.083 (0.899–4.827)	0.087	2.303 (0.831–6.381)	0.109
**Differentiation type(high or medium vs low)**	2.024 (0.599–6.846)	0.257	1.322 (0.421–4.153)	0.633
**Lauren's Classification (intestinal and mix type vs diffuse type)**	1.238 (0.529–2.896)	0.623	1.182 (0.428–3.260)	0.747
**Four-circRNA-based classifier (low vs high risk)**	6.248 (2.534–15.403)	0.000069	4.886 (1.375–17.359)	0.014
**Four-circRNA-based classifier plus (low vs high risk)**	22.156 (5.143–95.150)	0.000032	21.601 (2.830–164.902)	0.003

If we combined the circRNA classifier with traditional T and N stages, we could establish a new formula called circRNA classifier + TNM by using the following formula: new risk score = 2.709 × status of risk score (low risk as 0 and high risk as 1) + 1.432 × T stage + 0.678 × status of N stage. We assigned T2+T3 to 1, T4a to 2, and T4b to 3 and N0+N1 to 1, N2 to 2, N3a to 3, and N3b to 4. The cutoff score was “6” calculated by X-tile software. Patients were included in the high-risk group when their risk scores were higher than the cutoff score. Otherwise, patients were included in the low-risk group. The HRs of this formula were 22.156 (95% CI = 5.143–95.150, *p* = 0.000032) and 21.601 (95% CI = 2.830–164.902, *p* = 0.003) in the two cohorts, respectively. The areas under the ROC curve were 0.866 (95% CI = 0.769–0.962) and 0.818 (95% CI = 0.698–0.938) of the two cohorts, respectively. Their Harrell C-indices were 0.801 (95% CI = 0.665–0.937) and 0.780 (95% CI = 0.627–0.933), respectively. These results suggested that the proposed four-circRNA-based classifier could predict the early recurrence of stage III gastric cancer.

## DISCUSSION

Radical surgery is the most effective treatment for stage III gastric cancer [21]. However, early postoperative tumor recurrence is very difficult to manage, and this condition likely exacerbates a patient's prognosis. Therefore, we should develop new predictive factors that can identify early recurrence. This initiative mainly aims to categorize patients into high- or low-risk group. Thus, the most effective and least toxic treatment can be applied.

To our knowledge, this study is the first to identify a cluster of circRNA expressed in stage III gastric cancer. Our microarray results demonstrated that the expression of circRNAs in gastric carcinoma tissues was abnormal compared with adjacent normal tissues. For some of these indicators, the recurrence free survival rates of the postoperative patients were significantly different between the low and high expression groups. Moreover, we established a four-circRNA-based classifier that could divide the patients with stage III gastric cancer into low- or high-risk group of early recurrence. We can more accurately predict the patients’ recurrence rate by directly constructing the model of early recurrence than by using traditional classification indices, such as TNM staging and Lauren's typing. The ROC curve indicated that the predictive effect of our model was greater than that of traditional classification indices. The accuracy of the model can be further improved through its combination with traditional TNM staging.

CircRNAs affect cell functions through different pathways. CircRNAs form a sponge of miRNAs with its complementary sequences to reduce their function [17]. For example, circRNA CDR1 containing miR-7 binding sites can serve as a cancer development regulator by inhibiting miR-7 [22, 23], and circHIPK3 directly binds to miR-124 to inhibit human cell growth [24, 25]. circRNAs also perform other functions, such as RBP sponge and its regulator, mRNA regulator, or direct templates for protein synthesis [26]. Our study is the first to establish a four-circRNA-based classifier to predict the early recurrence for patients with stage III gastric cancer. In our clinical work, we can determine the possibility of the early recurrence of cancer patients by detecting circRNA expression in cancer tissues of patients after surgery. Thus, we can also confirm whether patients need a more intensive postoperative follow-up examination.

Our study is limited by several factors. The accuracy and specificity of the model cannot be estimated accurately because of the sample size. As such, our results should be further verified by conducting prospective and multicenter studies. The follow-up period of this research was 1 year, and no long-term survival data were available to explain the long-term predictive ability of the model. The correlation between long-term survival and selected circRNA should also be evaluated in future studies. Further functional experimental analyses of cancer cells should be carried out to determine whether these circRNA genes directly stimulate GC progression.

In conclusion, our study demonstrated that the expression profile of circRNAs in stage III gastric cancer was abnormal, and the proposed four-circRNA-based predictive tool could be more effective than current clinical predictors in the identification of patients with a high risk of early recurrence after radical surgery.

## MATERIALS AND METHODS

### Population

A total of 125 patients diagnosed with stage III gastric cancer were involved in this study. Fresh tissues were collected between December 2012 and May 2015 during radical surgery at Nanfang Hospital of Southern Medical University, frozen within 5 min in liquid nitrogen, and stored at −80°C. Cancer tissues were collected from the tumor surface, and the adjacent tissues were obtained from the normal intestinal epithelium at least 5 cm from the cancer tissues. None of the enrolled patients received chemotherapy, radiotherapy, or target therapy before they underwent radical surgery. After intervention was administered, all of the patients were subjected to standard six cycles of FOLFOX6 chemotherapy unless patients’ intolerance. No patient received biological therapy or immunotherapy. We used random numbers to assign 6 of these patients to microarray analysis, 67 patients were included in the training cohort and 52 patients were placed in the testing cohort. The baseline characteristics of patients are shown in Table 3. The workflow is illustrated in Supplementary Figure 2. The study protocol was approved by Institutional Review Board of Nanfang Hospital Southern Medical University. Informed consent was obtained from all of the patients involved in this study. All of the methods were performed in accordance with the relevant guidelines and regulations.

**Table 3:** Baseline characteristics of enrolled patients

		Training cohort		Testing cohort		Total	
		Number of patients	recurrence cases (%)	Number of patients	recurrence cases (%)	Number of patients	recurrence cases (%)
**Sex**	**Male**	45	17 (38%)	38	11 (29%)	83	28 (34%)
	**Female**	22	5 (23%)	14	4 (29%)	36	9 (25%)
**Age**	**65 years or younger**	58	18 (31%)	41	14 (34%)	99	32 (32%)
	**older than 65 years**	9	4 (44%)	11	1 (9%)	20	5 (25%)
**TNM stages**	**IIIA and IIIB**	35	7 (20%)	21	3 (14%)	56	10 (18%)
	**IIIC**	32	15 (47%)	31	12 (39%)	63	27 (43%)
**T stage**	**T2-T4a**	54	17 (31%)	40	10 (25%)	94	27 (29%)
	**T4b**	13	5 (38%)	12	5 (42%)	25	10 (40%)
**Number of nodes invasion examined**	**Less than 16**	40	10 (25%)	36	8 (22%)	76	18 (24%)
	**16 or more**	27	12 (44%)	16	7 (44%)	43	19 (44%)
**Differentiation type**	**high or medium**	15	3 (20%)	17	4 (24%)	32	7 (22%)
	**low**	51	19 (37%)	35	11 (31%)	86	30 (35%)
**Lauren's Classification**	**Intestinal and mixed type**	31	9 (29%)	26	7 (27%)	57	16 (28%)
	**Diffuse type**	36	13 (34%)	26	8 (31%)	62	21 (34%)

### Total RNA extraction

Frozen tissues (20–50 mg) were homogenized, and Trizol reagent (Takara, Japan) was used to extract total RNA from tissues according to the manufacturer's instructions.

### Circular RNA microarray

Human circRNA microarray 1.0 (Arraystar, USA) containing 5396 circular RNA probes was used. CircRNA was purified from the total RNA by using RNase R. In the six-paired cases, two cases developed recurrence within 1 year and the four other cases did not experience recurrence.

### qRT-PCR and construction of the circRNA signature classifier

cDNA was synthesized through reverse transcription by using GoScript RT System (Promega, USA). Real-time quantitative reverse transcription polymerase chain reaction (qRT-PCR) was conducted with Go*Taq* qPCR Master Mix (Promega, USA). The thermocycler programs were as follows: 95°C for 10 min and 40 cycles of 95°C for 30 sec, 55°C annealing temperature for primer pairs for 30 sec, and 72°C for 30 sec. The PCR products were separated on a 2% agarose gel, stained with Gelview, and visualized under ultraviolet illumination. Each reaction was performed in triplicate. Reverse primers were designed to ensure that circRNAs were amplified through head-to-tail splicing [13]. The primer sequences of the selected circular RNA and GADPH are shown in Supplementary Table 2.

The optimum cutoff point for the circRNA expression level of the high and low expression groups for the expressed circRNAs in the training set was calculated by using X-tile software. This software can automatically provide the optimum point of segmentation based on a patient's survival defined by Kaplan–Meier survival analysis [27]. Univariate cox regression analysis was carried out to select the circRNA signatures, and these signatures were verified through multivariate cox regression analysis. A formula was subsequently derived to calculate the risk score of the recurrence probability based on the individual expression level of the four circRNAs by using Cox regression model.

### Statistical analysis

In microarray analysis, circRNAs were identified as differentially expressed between tumor tissues and adjacent mucosal tissues if the expression change was 3-fold or more with a *p*-value of < 0.05. CircRNAs between recurrence cases and other cases were classified as differentially expressed if the expression change was 3-fold or more. In qRT-PCR, the expression level of circRNA was calculated as mean ΔCT (CT value of target − CT value of GADPH). X-tile 3.6.1 software (Yale University, USA) was then used to obtain the optimum cutoff point of the circRNA expression level of high and low expression groups for the expressed circRNAs [28]. SPSS 20.0 was utilized to calculate HR and draw the ROC curve. R software 2.11.1 was employed to calculate Harrell C-index.

## SUPPLEMENTARY MATERIALS FIGURES AND TABLES



## Abbreviations

circRNA: circular RNA
HR: hazard ratio
AUC: area under curve
ROC: receiver operator characteristic

## References

[1] Torre LA, Bray F, Siegel RL, Ferlay J, Lortet-Tieulent J, Jemal A (2015). Global cancer statistics, 2012. CA Cancer J Clin.

[2] Ferlay J, Soerjomataram I, Dikshit R, Eser S, Mathers C, Rebelo M, Parkin DM, Forman D, Bray F (2015). Cancer incidence and mortality worldwide: sources, methods and major patterns in GLOBOCAN 2012. Int J Cancer.

[3] Washington K (2010). edition of the AJCC cancer staging manual: stomach. Ann Surg Oncol.

[4] Yang K, Choi YY, Zhang WH, Chen XZ, Song MK, Lee J, Zhang B, Chen ZX, Kim HI, Chen JP, Cheong JH, Zhou ZG, Hyung WJ (2016). Strategies to improve treatment outcome in gastric cancer: A retrospective analysis of patients from two high-volume hospitals in Korea and China. Oncotarget.

[5] Spolverato G, Ejaz A, Kim Y, Squires MH, Poultsides GA, Fields RC, Schmidt C, Weber SM, Votanopoulos K, Maithel SK, Pawlik TM (2014). Rates and patterns of recurrence after curative intent resection for gastric cancer: a United States multi-institutional analysis. J Am Coll Surgeons.

[6] Qi X, Liu Y, Wang W, Cai D, Li W, Hui J, Liu C, Zhao Y, Li G (2016). Management of advanced gastric cancer: An overview of major findings from meta-analysis. Oncotarget.

[7] Wilusz JE, Sharp PA (2013). Molecular biology. A circuitous route to noncoding RNA. Science.

[8] Salzman J, Gawad C, Wang PL, Lacayo N, Brown PO (2012). Circular RNAs are the predominant transcript isoform from hundreds of human genes in diverse cell types. PloS One.

[9] Chen L, Huang C, Wang X, Shan G (2015). Circular RNAs in Eukaryotic Cells. Curr Genomics.

[10] Chen I, Chen CY, Chuang TJ (2015). Biogenesis, identification, and function of exonic circular RNAs. WIRES RNA.

[11] Jeck WR, Sorrentino JA, Wang K, Slevin MK, Burd CE, Liu J, Marzluff WF, Sharpless NE (2013). Circular RNAs are abundant, conserved, and associated with ALU repeats. Rna.

[12] Jeck WR, Sharpless NE (2014). Detecting and characterizing circular RNAs. Nat Biotechnol.

[13] Memczak S, Jens M, Elefsinioti A, Torti F, Krueger J, Rybak A, Maier L, Mackowiak SD, Gregersen LH, Munschauer M, Loewer A, Ziebold U, Landthaler M (2013). Circular RNAs are a large class of animal RNAs with regulatory potency. Nature.

[14] Zhang YG, Yang HL, Long Y, Li WL (2016). Circular RNA in blood corpuscles combined with plasma protein factor for early prediction of pre-eclampsia. BJOG-Int J Obstet Gy.

[15] Wang X, Zhang Y, Huang L, Zhang J, Pan F, Li B, Yan Y, Jia B, Liu H, Li S, Zheng W (2015). Decreased expression of hsa_circ_001988 in colorectal cancer and its clinical significances. Int J Clin exp Patho.

[16] Qin M, Liu G, Huo X, Tao X, Sun X, Ge Z, Yang J, Fan J, Liu L, Qin W (2016). Hsa_circ_0001649: A circular RNA and potential novel biomarker for hepatocellular carcinoma. Cancer Biomark.

[17] Xuan L, Qu L, Zhou H, Wang P, Yu H, Wu T, Wang X, Li Q, Tian L, Liu M, Sun Y (2016). Circular RNA: a novel biomarker for progressive laryngeal cancer. Am J Transl Res.

[18] Song X, Zhang N, Han P, Moon BS, Lai RK, Wang K, Lu W (2016). Circular RNA profile in gliomas revealed by identification tool UROBORUS. Nucleic acids research.

[19] Li Y, Zheng Q, Bao C, Li S, Guo W, Zhao J, Chen D, Gu J, He X, Huang S (2015). Circular RNA is enriched and stable in exosomes: a promising biomarker for cancer diagnosis. Cell Res.

[20] Guarnerio J, Bezzi M, Jeong JC, Paffenholz SV, Berry K, Naldini MM, Lo-Coco F, Tay Y, Beck AH, Pandolfi PP (2016). Oncogenic Role of Fusion-circRNAs Derived from Cancer-Associated Chromosomal Translocations. Cell.

[21] Tegels JJ, De Maat MF, Hulsewe KW, Hoofwijk AG, Stoot JH (2014). Improving the outcomes in gastric cancer surgery. World J Gastroentero.

[22] Xu H, Guo S, Li W, Yu P (2015). The circular RNA Cdr1as, via miR-7 and its targets, regulates insulin transcription and secretion in islet cells. Sci Rep.

[23] Zhao ZJ, Shen J (2015). Circular RNA Participates in the Carcinogenesis and the Malignant Behavior of Cancer. RNA Biology.

[24] Zheng Q, Bao C, Guo W, Li S, Chen J, Chen B, Luo Y, Lyu D, Li Y, Shi G, Liang L, Gu J, He X (2016). Circular RNA profiling reveals an abundant circHIPK3 that regulates cell growth by sponging multiple miRNAs. Nat Commun.

[25] Qi X, Zhang L, Lu X (2016). New insights into epithelial-to-mesenchymal transition in cancer. Trends Pharmacol Sci.

[26] Hentze MW, Preiss T (2013). Circular RNAs: splicing's enigma variations. EMBO J.

[27] Camp RL, Dolled-Filhart M, Rimm DL (2004). X-tile: a new bio-informatics tool for biomarker assessment and outcome-based cut-point optimization. Clin Cancer Res.

[28] Jiang Y, Zhang Q, Hu Y, Li T, Yu J, Zhao L, Ye G, Deng H, Mou T, Cai S, Zhou Z, Liu H, Chen G (2016). ImmunoScore Signature: A Prognostic and Predictive Tool in Gastric Cancer. Ann Surg.

