# Supplementary material for: Circular RNAs signature predicts the early recurrence of stage III gastric cancer after radical surgery
Source: Oncotarget. 2017 Feb 11;8(14):22936–43. doi: 10.18632/oncotarget.15288 (PMC5410275; doi:10.18632/oncotarget.15288)
Supplement: Supplementary file 1 [file oncotarget-08-22936-s001.pdf]

Circular RNAs signature predicts the early recurrence of stage III gastric cancer after radical surgery

Supplementary Materials

Supplementary Table 1: Univariate and multivariate analysis of circRNAs with recurrence free survival in training cohort

Parameters	categoris(ΔCT value)	Univariate analysis		Multivariate analysis	
		HR(95%CI)	*P* value	HR(95%CI)	*P* value
circRNA_101308	Low (≤– 9.09) vs. High expression (>– 9.09)	2.717(1.717–6.297)	0.02	4.197(1.644–10.712)	0.003
circRNA_001653	Low (≤– 10.36) vs. High expression (>– 10.36)	0.602(0.261–1.389)	0.234		
circRNA_103781	Low (≤– 9.7) vs. High expression (>– 9.7)	0.614(0.266–1.448)	0.253		
circRNA_104423	Low (≤– 12.24) vs. High expression (>– 12.24)	0.318(0.137–0.739)	0.008	0.400(0.165–0.971)	0.043
circRNA_103999	Low (≤– 8.81) vs. High expression (>– 8.81)	0.483(0.203–1.153)	0.101		
circRNA_104916	Low (≤– 10.26) vs. High expression (>– 10.26)	0.276(0.118–0.647)	0.003	0.353(0.143–0.870)	0.024
circRNA_100269	Low (≤– 11.51) vs. High expression (>– 11.51)	0.355(0.154–0.821)	0.016	0.301(0.121–0.749)	0.01

Supplementary Table 2: Primer sequences for qRT-PCR

Gene	Primer sequence
**hsa_circRNA_103999**	F:5′ATTGCAGATGCCTTCAACCTC3′<br>R :5′CTTCCTTTGCTAAATTCCCAGA3′
**hsa_circRNA_103781**	F:5′AGCAACCTCATCACCTGGAAC3′<br>R :5′TGTCTCTTCCTCCAGATTTTCACT3′
**hsa_circRNA_104423**	F:5′CCACTGGCAAAGAGTCACCTAAA3′<br>R :5′ATTCCCTGGCAGTTCCGTGTA3′
**hsa_circRNA_001653**	F:5′GATGTTGCCCAGTGGCTGT3′<br>R :5′AGGGGCAGCAAGCTCAGGT3′
**hsa_circRNA_101308**	F:5′ CACCTCCATCGAACCCATCC3′<br>R:5′ CAAGCCAAATGCAATCATTAACAG3′
**hsa_circRNA_104916**	F:5′ GCTCGGTGACCTTGGTCTGG3′<br>R:5′ GCGTGTTGGGATGCCTCTGT3′
**hsa_circRNA_100269**	F:5′ CTAACTATGGTCGGACGGATGA 3′<br>R:5′ CAATGATAAACCACAGACTTCGC3′
**hsa_circRNA_400033**	F:5′ TTCGCCTCTGGTATCGTGC3′<br>R:5′ GTTCCCAATTCGTTCGCTCT 3′
**GAPDH**	F:5′GGGAAACTGTGGCGTGAT3′<br>R:5′GAGTGGGTGTCGCTGTTGA3′

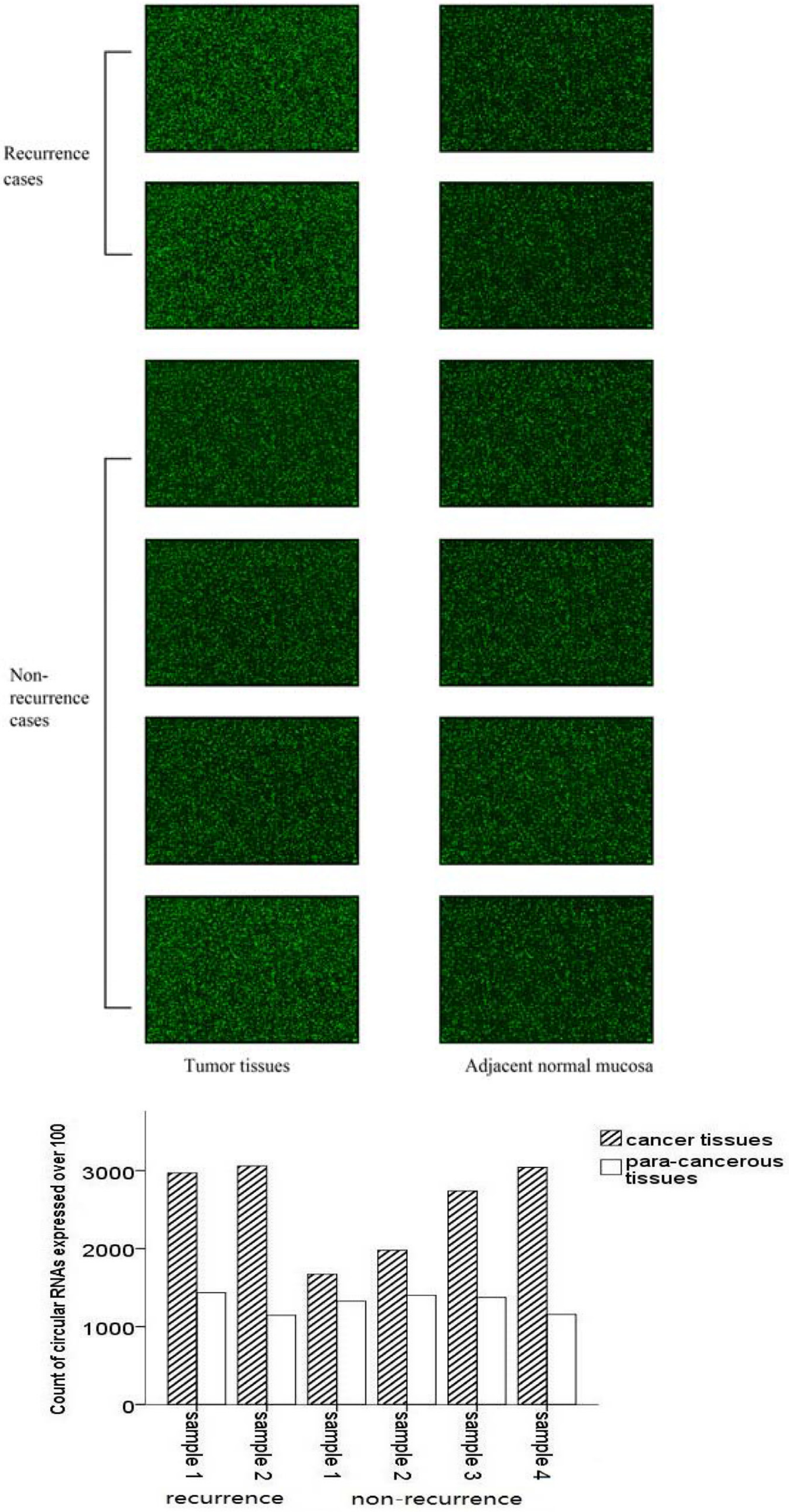

Supplementary Figure 1: Raw graphs of circRNA microarray, six tissue samples on the left are the cancer tissues, six on the right side are normal para-cancerous tissues; among the six cases, three patients developed recurrence within one year, the other three cases was found no recurrence within one year; circular RNAs were founded over expressed in cancer tissues (the count of circular RNA expressed over 100 in cancer tissues were much than para-cancerous tissues)

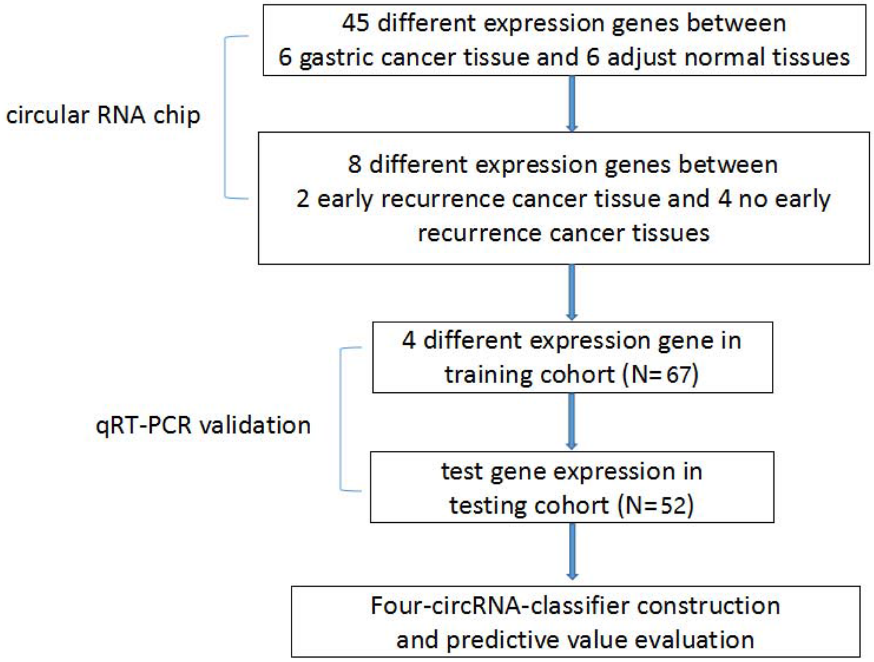

Supplementary Figure 2: Workflow of the experiment, we conducted circular RNA chip of 6 gastric cancer tissue comparing adjust normal gastric mucosa tissues, and we carried out the RT-qPCR validation of the final 8 indicators, then we constructed a four-circRNA-combined classifier using linear regression model.
